# Inactivation of SARS-CoV-2 and photocatalytic degradation by TiO_2_ photocatalyst coatings

**DOI:** 10.1038/s41598-022-20459-2

**Published:** 2022-09-26

**Authors:** Yun Lu, Sujun Guan, Liang Hao, Hiroyuki Yoshida, Shohei Nakada, Taisei Takisawa, Takaomi Itoi

**Affiliations:** 1 Department of Mechanical Engineering, Chiba University, Chiba, 2638522 Japan; 2 Bio-Nano Electronics Research Centre, Toyo University, Saitama, 3508585 Japan; 3 College of Mechanical Engineering, Tianjin University of Science and Technology, Tianjin, 300222 China; 4 Chiba Industrial Technology Research Institute, Chiba, 2640017 Japan

**Keywords:** Photocatalysis, Pollution remediation

## Abstract

The novel severe acute respiratory syndrome coronavirus 2 (SARS-CoV-2) causative agent of the COVID-19, which is a global pandemic, has infected more than 552 million people, and killed more than 6.3 million people. SARS-CoV-2 can be transmitted through airborne route in addition to direct contact and droplet modes, the development of disinfectants that can be applied in working spaces without evacuating people is urgently needed. TiO_2_ is well known with some features of the purification, antibacterial/sterilization, making it could be developed disinfectants that can be applied in working spaces without evacuating people. Facing the severe epidemic, we expect to fully expand the application of our proposed effective approach of mechanical coating technique (MCT), which can be prepared on a large-scale fabrication of an easy-to-use TiO_2_/Ti photocatalyst coating, with hope to curb the epidemic. The photocatalytic inactivation of SARS-CoV-2 and influenza virus, and the photocatalytic degradation of acetaldehyde (C_2_H_4_O) and formaldehyde (CH_2_O) has been investigated. XRD and SEM results show that anatase TiO_2_ successfully coats on the surface of Ti coatings, while the crystal structure of anatase TiO_2_ can be increased during the following oxidation in air. The catalytic activity towards methylene blue of TiO_2_/Ti coating balls has been significantly enhanced by the followed oxidation in air, showing a very satisfying photocatalytic degradation of C_2_H_4_O and CH_2_O. Notably, the TiO_2_/Ti photocatalyst coating balls demonstrate a significant antiviral activity, with a decrease rate of virus reached 99.96% for influenza virus and 99.99% for SARS-CoV-2.

## Introduction

From 2019, SARS-CoV-2 is a novel pathogenic human coronavirus that led to an atypical pneumonia-like severe acute respiratory syndrome (SARS) outbreak called COVID-19, which had a direct blow to people's life, society, mobility, and globalization in the recent times, and will have an unprecedented impact on modern human civilization in the long term. In general, SARS-CoV-2 could be transmitted rapidly via contaminated surfaces and aerosols, emphasizing the importance of environmental disinfection to block the spread of virus^1–3^. Even worse, SARS-CoV-2 is believed to be transmitted through the way of airborne route except for the generally recognized direct-contact and droplet modes^4,5^. Facing to the global pandemic, ultraviolet (UV) C radiation and chemical compounds are first thought to apply to the disinfection of SARS-CoV-2, with necessary contactless space with humans to avoid their toxicities^2^. Therefore, the development of disinfectants that can be applied in working spaces without evacuating people is urgently needed. In 2021, a study demonstrated for the first time that a titanium oxide (TiO_2_) photocatalyst-coated glass sheet could inactivate 99.9% of SARS-CoV-2 in aerosols in 20 min, due to the photocatalytic damage to SARS-CoV-2 virus particles, RNA damage, and degradation of viral proteins^6^. In addition, 2 ml of virus solution was dropped onto a 3 cm square photocatalyst-coated glass sheet, and the virus in the liquid was inactivated at the rate of 99.9% in 120 min by visible light at 405 nm. Recently, Nakano et al. reported that copper oxide nanoclusters grafted onto rutile TiO_2_ powder can effectively inactivate SARS‐CoV‐2 virus, even under dark condition or illumination with a white fluorescent bulb^7^.

Owing to the robustness and feasibility for use as coating materials, the solid-state antiviral compounds are expected to be useful in inactivating viruses on a large scale. Among the current various solid-state antiviral compounds, TiO_2_ photocatalyst is promising because of their Earth-rich, non-toxic, chemically stability, and higher antiviral effect under UV, visible light, and near-infrared light irradiation^8–13^. In addition, after the so-called Honda-Fujishima effect for the water splitting using TiO_2_ photocatalyst^8^, widely deployed from basic research to application technology development, focusing, and antibacterial/sterilization, self-cleaning, energy-saving air conditioning, and so on^14–16^. Various volatile organic compounds (VOCs) such as acetaldehyde, benzene and formaldehyde are considered as air toxins and known for their adverse effects on health, and the VOCs could be subject to disruption by an oxidation process, caused by TiO_2_ photocatalyst^17–20^. In 2021, Xie et al. demonstrated that intermediates accumulation was primarily responsible for the deactivation of the TiO_2_ photocatalyst, which expected to overcome the fundamental issues to be addressed for photodegrading VOCs in practical applications caused by poor efficiency and stability of photocatalysts^18^.

Herein, this study demonstrates the inactivation of SARS-CoV-2 and photocatalytic degradation of C_2_H_4_O and CH_2_O, with the presented TiO_2_/Ti photocatalyst coatings, formed on 2 mm diameter Al_2_O_3_ balls. Notably, the TiO_2_/Ti photocatalyst coatings balls show a very satisfying photocatalytic degradation of C_2_H_4_O and CH_2_O, and a significant inactivation towards influenza virus and SARS-CoV-2.

## Experimental method

### Fabrication of TiO_2_/Ti photocatalyst coatings on Al_2_O_3_ balls

TiO_2_/Ti photocatalyst coatings were formed on Al_2_O_3_ balls using the previously established MCT^21–23^. First, Al_2_O_3_ balls (approximately 2 mm diameter) and Ti powder (particle size less than 45 µm, purity 99.4%) were filled in sequence into an alumina pot, with a covered alumina lid. The Ti coatings were formed on Al_2_O_3_ balls by MCT, with a planetary ball mill (P-6; FRITSCH) at a rotational speed of 480 rpm for 3 h, named as "Ti". Then, TiO_2_ photocatalyst coatings were formed on the Ti coatings by MCT, with filling the Ti sample and TiO_2_ powder (ST-01, particle size of 7 nm) in an alumina pot at a MCT rotational speed of 300 rpm for 3 h, named as "TiO_2_/Ti". To enhance the photocatalytic activity of the coatings, the TiO_2_/Ti sample were subjected to heat treatment at 500 °C for 5 h in air using an electric furnace. After the oxidation, the sample is marked as "TiO_2_/Ti–O".

### Characterization of TiO_2_/Ti photocatalyst coatings on Al_2_O_3_ balls

The crystal structure of the fabricated TiO_2_/Ti photocatalyst coatings were analyzed by an X-ray diffractometer (XRD, Rigaku Ultima IV) with Cu-Kα radiation, the surface and cross-section were observed by a scanning electron microscopy (SEM, Hatachi-8030). X-ray photoelectron spectroscopy (XPS, PHI Quantes) measurements was used to observe the change in the chemical composition on the surface. According to ISO 10678-2010, a wet decomposition performance test under UV irradiation towards methylene blue (MB) was used to evaluate the photocatalytic function of the TiO_2_/Ti photocatalyst coatings on Al_2_O_3_ balls. The test cells (inner diameter Φ 40 mm × 30 mm, cylindrically shaped with a bottom) were spread over one-layer sample and filled with MB solution (20 μmol/L, 35 mL), then eliminated any possible absorption by keep the cells in dark for 18 h for adsorption. Followed by the adsorption, the cells were refreshed with a test MB solution (10 μmol/L, 35 mL) and irradiated with UV light of 1.0 mW/cm^2^ intensity for 3 h. The absorbance of the MB solution at 640 nm was measured within every 20 min using a digital colorimeter (mini photo 10; Sanshin Kogyo).

### Evaluation tests of the environmental purification

The environmental function evaluation test was conducted by the Kanagawa Institute of Industrial Science and Technology, a public research institute. Acetaldehyde (C_2_H_4_O), which causes sick house syndrome, etc. due to its odor and irritation, and formaldehyde (CH_2_O), a toxic substance that causes inflammation of the human respiratory system, eyes, and throat, contained in adhesives used in building materials such as furniture and wallpaper, were used as the targets. The decomposition and removal performance tests of C_2_H_4_O and CH_2_O were conducted at approximately 25 °C, as per JIS R 1701-2:2016 (Testing methods for air purification performance of fine ceramics-Photocatalytic materials—Part 2: Removal performance of acetaldehyde) and JIS R 1701-4:2016 (Fine ceramics—Air purification performance test method for photocatalytic materials-Part 4: Formaldehyde removal performance), with spreading the TiO_2_/Ti–O sample over a 100 × 50 mm cell to be a single layer. For the decomposition and removal performance test of C_2_H_4_O, the concentration of the target gas was 5 ppm at a flowing rate of 1.0 L/min, and the UV irradiation was 10 W/m^2^, then the C_2_H_4_O concentration and CO_2_ concentration were measured. While the CH_2_O decomposition removal performance test, the concentration of test gas was set to 1.02 ppm at a flowing rate of 3.0 L/min, and the UV irradiation to 1.0 mW/cm^2^.

### Inactivation performance tests

In compassion of the currently inactivation of new coronaviruses by sheet and plate photocatalysts, we tested the inactivation performance of influenza virus and SARS-CoV-2 on TiO_2_/Ti photocatalytic coatings on balls. The inactivation test of influenza virus was conducted by requesting a test from the Kanagawa Institute of Industrial Science and Technology. The tests were conducted at approximately 25 °C, according to JIS R 1706:2020 (UV-responsive photocatalyst, antiviral, film adhesion method). Influenza A virus (H3N2) was used as the viral strain, ATCC CCL-34 as the host cell, and the irradiation conditions were UV irradiation of 0.25 mW/cm^2^ with a black light fluorescent lamp, or 0 mW/cm^2^ (in dark). The samples were sterilized and pre-irradiated with UV rays at 1.0 mW/cm^2^ for 24 h, then aseptic treated at 80 °C for 15 min. The samples were spread in a sterile petri dish with a diameter of 60 mm to form a single layer. Then, 2.4 ml of sterile water and 0.1 ml of the virus solution were added and covered with a glass plate for moisture retention. After 8 h of UV irradiation and storage in dark, the infectivity titer of the virus was determined by the plaque method.

Furthermore, the inactivation test of SARS-CoV-2 was conducted according to JIS R 1706 (Test method for antiviral activity of fine ceramic photocatalytic materials), at Nara Medical University. Infected Vero E6 cells with SARS-CoV-2 were used as the target, stored in a − 80 °C freezer before the test. The UV irradiation conditions were 0.25 mW/cm^2^ with a black light fluorescent lamp, or 0 mW/cm^2^ (in dark). After the operation time, viruses were recovered with phosphate-buffered saline (PBS) solution. The cells were observed after 3 days of incubation, and viral infection titer and viral inactivation effects were calculated.

## Results and discussion

### Characterizations and photocatalytic activity of TiO_2_/Ti photocatalyst coatings on Al_2_O_3_ balls

**Figure 1:**
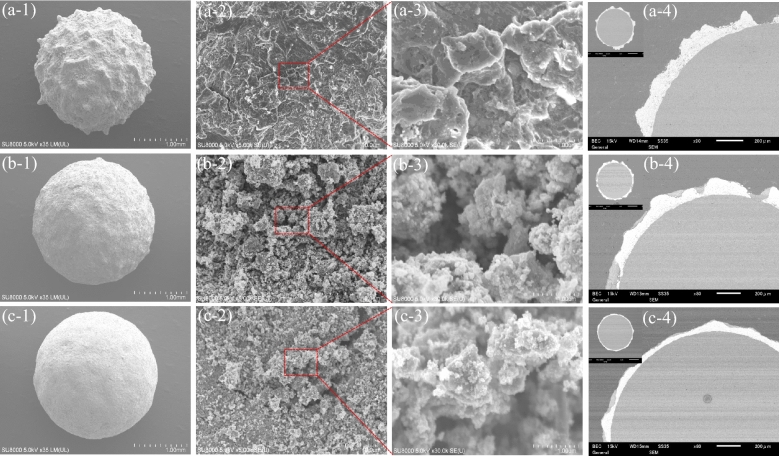
SEM microstructures of the surfaces and cross sections of the samples. (**a**) Ti, (**b**) TiO_2_/Ti, and (**c**) TiO_2_/Ti–O.

**Figure 2:**
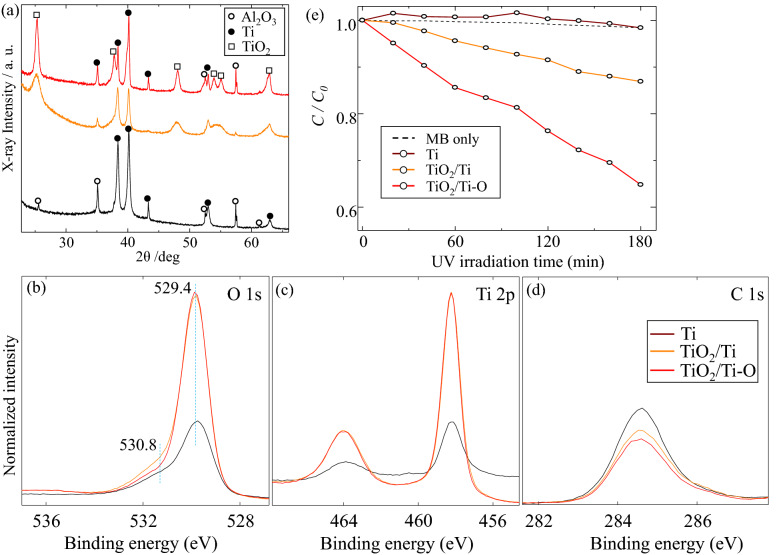
XRD patterns of the samples. (**a**) XRD patterns, (**b**) O 1s XPS spectra, (**c**) Ti 2p XPS spectra, (**d**) C 1s XPS spectra, and (**e**) the photocatalytic activity towards the degradation of MB solution.

**Figure 3:**
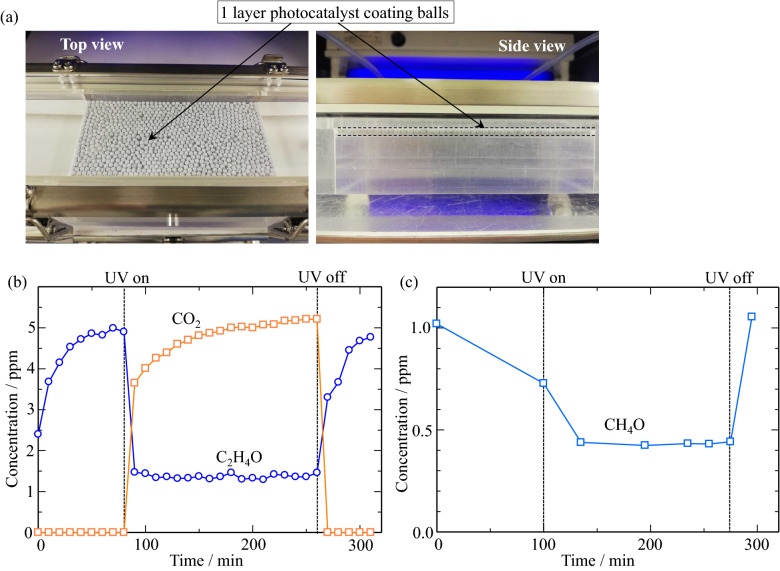
The decomposition test of C_2_H_4_O and CH_2_O with the TiO_2_/Ti–O sample. (**a**) the set-up, (**b**) the concentration changes of C_2_H_4_O and CO_2_, (**c**) the concentration changes of CH_2_O.

**Figure 4:**
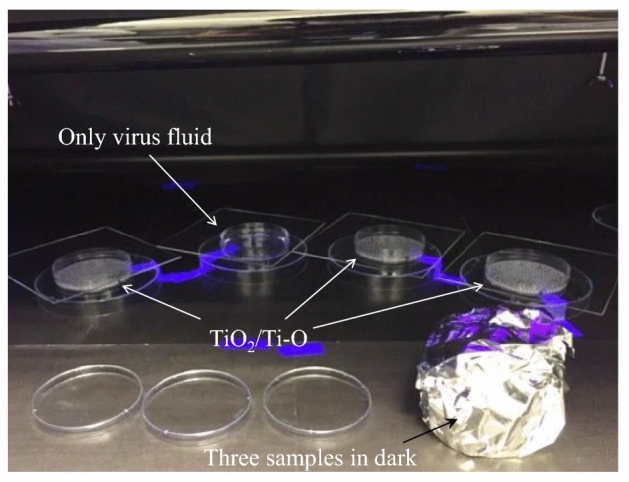
The set-up of antiviral test by using influenza virus under UV irradiation and in dark.

The appearance photographs of the Al_2_O_3_ balls (2 mm diameter), and the samples of Ti and TiO_2_/Ti. Ti coatings and TiO_2_/Ti coatings are presented in Fig. S1. The Ti coatings and TiO_2_ coatings have been formed on the surface of the Al_2_O_3_ balls, due to the change in color and appearance, which is similar to those of 1 mm Al_2_O_3_ balls so date^21^. The surface and cross-sectional SEM images of the samples of Ti, TiO_2_/Ti, and TiO_2_/Ti–O are shown in Fig. 1. It could find that the formed Ti coatings are a bulge-like structure (Fig. 1a-1) and uneven (Fig. 1a-4), compared with that of Al_2_O_3_ balls (Fig. S2). Then, the TiO_2_ coatings formed on the surface of the Ti coatings show grainy textured surface structure (Fig. 1b-2). Interesting, the uneven part of the Ti coatings has been filled with TiO_2_ coatings (Fig. 1b-4), which make the surface to be smooth (Fig. 1b-1). In addition, the thicknesses of the Ti and TiO_2_ coatings are approximately 97 μm and 3 μm, respectively, according to the abbreviated calculations from SEM photographs. However, with comparison of the samples of TiO_2_/Ti and TiO_2_/Ti–O, the influence of followed oxidation in air at 500 °C for 5 h on the surface structure and cross sections is insignificant. Figure 2a shows the XRD patterns of the samples of Ti, TiO_2_/Ti, and TiO_2_/Ti–O. In general, the Ti peaks and TiO_2_ peaks mean that Ti coatings and TiO_2_ coatings successfully form on Al_2_O_3_ balls. After oxidation in air, the Al_2_O_3_ peaks disappear and the Ti and anatase TiO_2_ peaks significantly increase, which indicates that the crystallinity of anatase TiO_2_ has been greatly enhanced.

XPS spectra has been used to investigate the change of chemical bonding on the surface of the samples, as shown in Fig. 2b–d. For comparison, Fig. 2b shows the O 1 s peak at around 529.4 eV of the samples, which could be corresponded to the Ti–O bonding from the anatase TiO_2_^24,25^. Although the O 1 s shift hardly could be found from the samples of TiO_2_/Ti and TiO_2_/Ti–O, but the peak at around 530.8 eV from the TiO_2_/Ti–O sample decrease, compared with that of the TiO_2_/Ti–O sample, which hints that the crystallinity of anatase TiO_2_ has been greatly enhanced, matching with the XRD results. Figure 2e reveals that the samples of TiO_2_/Ti and TiO_2_/Ti–O exhibit excellent photocatalytic activity, compared with that of Ti coatings. In general, the TiO_2_ coatings clearly shows the photocatalytic activity, and the photocatalytic activity could be further enhanced with an increased crystallinity of anatase TiO_2_.

### Environmental purification function of the TiO_2_/Ti–O sample on Al_2_O_3_ balls

Figure 3 shows the decomposition and removal performance of the TiO_2_/Ti–O sample for C_2_H_4_O. The set-up of the decomposition performance for C_2_H_4_O and one layer of the TiO_2_/Ti–O sample are presented in Fig. 3a. The concentration of C_2_H_4_O increases from the beginning of the test and reaches to 5 ppm, as shown in Fig. 3b. When UV light turns on, the concentration of C_2_H_4_O decrease rapidly and remains at about 1.3 ppm due to the decomposition by the TiO_2_/Ti–O sample on Al_2_O_3_ balls. In addition, the CO_2_ concentration generates by the decomposition of C_2_H_4_O^26–28^, and increases with the decomposition progresses. While the UV irradiation turns off, the concentration of C_2_H_4_O returns to nearly 5 ppm of the supply concentration, and the CO_2_ concentration decreases to 0 ppm again. The results mean that the decomposition function of the TiO_2_/Ti–O sample for C_2_H_4_O is significant and efficient. In general, when TiO_2_ has been illuminated with photons having energy higher than its bandgap, the electrons and holes will be simultaneously generated then separated to conduction band and valence band, respectively. The charge carriers can migrate to the surface of the photocatalyst and react with O_2_, H_2_O or hydroxyl groups, with generating OH^⋅^ and O_2_^−⋅^. During the decomposition, C_2_H_4_O has been firstly adsorbed on the surface of the TiO_2_ photocatalyst. Then, a part of C_2_H_4_O could be oxidized into CO_2_ and H_2_O by O_2_^−⋅^ or OH^⋅^ directly. The rest could firstly be oxidized into acetic acid by OH^⋅^, and then oxidized into CO_2_ and H_2_O by O_2_^−⋅^^27,28^.

Furthermore, Fig. 3c shows the decomposition and removal performance of the TiO_2_/Ti–O sample for CH_2_O. When UV light turns on, the concentration of CH_2_O rapidly decreases from 1 ppm, then keeps approximately 0.43 ppm. It has believed that the formed hydroxyl radicals transfer on the surface of TiO_2_ can not only directly react with CH_2_O molecules, but also can suppress the recombination of electron–hole during the transfer process to further enhance the photocatalytic activity^29–31^. When the UV light stops, the concentration of CH_2_O quickly returns to 1 ppm of the supplied concentration. These results also reveal the high decomposition ability of the TiO_2_/Ti–O sample for CH_2_O. In the case of the degradation process of CH_2_O, the generated OH^⋅^ and O_2_^−⋅^ will firstly attack the C–H bonds in CH_2_O, then react with the liberated hydrogen atoms to form new free radicals^29,30^. In general, the initial stage of the degradation process will produce formic acid, then ultimately decompose CH_2_O molecules into H_2_O and CO_2_.

### Virus inactivation by the TiO_2_/Ti–O sample on Al_2_O_3_ balls

Figure 4 shows the setup of inactivation test for influenza virus of H3N2, according to JIS R 1706:2020. Table 1 shows the infectious value and antiviral activity value of the samples under UV irradiation and in dark. The antiviral activity values are calculated by the following equations of (1) and (2).

1 $${\text{Antiviral activity value }}\left( {\text{bright spot}} \right){:}\;{\text{ V}}_{{\text{L}}} = {\text{ Log}}\left( {{\text{B}}_{{\text{L}}} } \right) \, - {\text{ Log}}\left( {{\text{C}}_{{\text{L}}} } \right)$$

2 $${\text{Antiviral activity value }}\left( {{\text{dark}}} \right){:}\;{\text{ V}}_{{\text{D}}} = {\text{ Log}}\left( {{\text{B}}_{{\text{D}}} } \right) \, - {\text{ Log}}\left( {{\text{C}}_{{\text{D}}} } \right)$$

where B is the infection titer of virus solution only, C is the infection titer of specimen, L is with UV irradiation, and D is in dark. According to ISO 18184 Annex G, an antiviral activity value of 3.0 or higher is considered effective antiviral activity, therefore, an average value of V_0.25_ = 3.4 from the TiO_2_/Ti–O sample is sufficient for antiviral effectiveness. The virus inactivation rate calculated from the average infectious value of 87 pfu/ml under 0.25 mW/cm^2^ reaches 99.96%, indicating that the TiO_2_/Ti–O sample have very high inactivation function for influenza virus.

**Table 1:** The infectious value and antiviral activity value of the samples, under UV irradiation (0.25 mW/cm^2^) and in dark.

Sample	Infectious value under UV (pfu/ml)	Antiviral activity value	Infectious value in dark (pfu/ml)	Antiviral activity value
Only virus fluid	2.0 × 10^5^	–	–	–
TiO_2_/Ti–O	–	–	1.9 × 10^4^	1.0
	–	–	2.0 × 10^4^	1.0
	–	–	2.6 × 10^4^	0.9
TiO_2_/Ti–O	< 10	4.3	–	–
	4.0 × 10^1^	3.7	–	–
	2.1 × 10^2^	3.0	–	–
Average value	8.7 × 10^1^	3.4	2.2 × 10^4^	1.0

**Figure 5:**
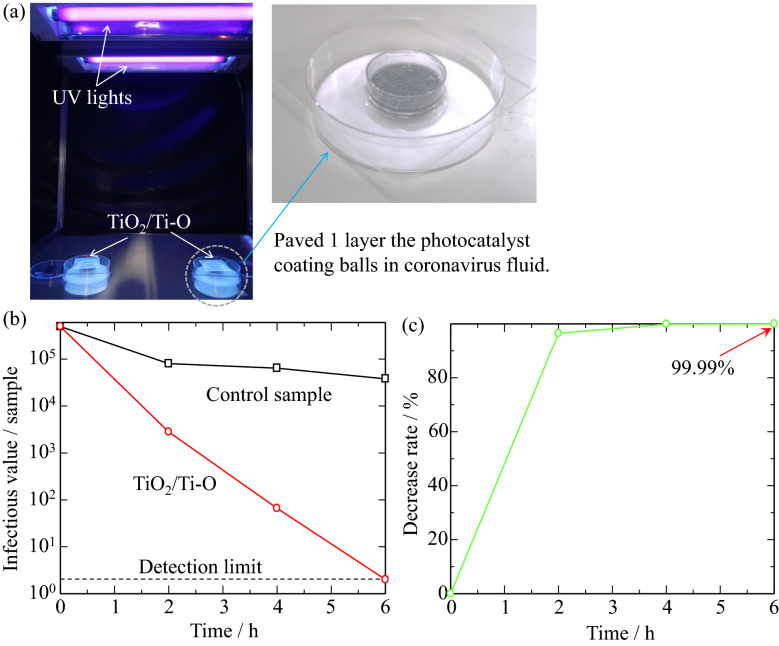
The inactivation test for SARS-CoV-2 of the TiO_2_/Ti–O sample. (**a**) the set-up, (**b**) the infectious value change of SARS-CoV-2, (**c**) the decrease rate of SARS-CoV-2.

Figure 5 shows the inactivation test of the TiO_2_/Ti–O sample on Al_2_O_3_ balls for SARS-CoV-2. Figure 5a clearly shows the setup of inactivation test. The infection titer of the control under UV light irradiation tends to decrease, whereas the infection titer of the TiO_2_/Ti–O sample significantly decreases, with an infectious value below than the detection limit after 6 h, as shown in Fig. 5b. In addition, the decrease rate of virus has been calculated and shown in Fig. 5c. The inactivation function of the TiO_2_/Ti–O sample is satisfactory in UV irradiation, and the decrease virus rate rapidly increases to 96% in short time, with reaching 99.99% within 6 h. These results mean that the TiO_2_/Ti–O sample are with a high inactivation function against the SARS-CoV-2.

**Figure 6:**
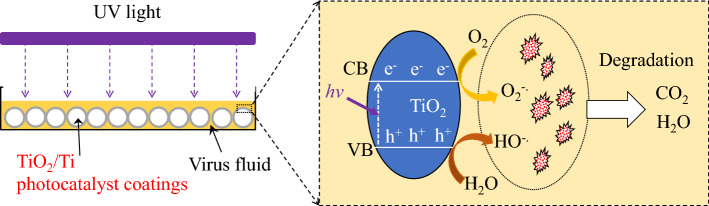
Proposed mechanisms of viral inactivation induced by TiO_2_/Ti photocatalyst coatings.

It is well known that TiO_2_ is a semiconductor metal oxide photocatalyst with a wide band gap of 3.2 eV (anatase type)^32^. TiO_2_ when exposed to UV light of energy equal to or greater than its band gap, there is an excitation of electrons from valance band (VB) to conduction band (CB) of TiO_2_. These charge carriers move onto the surface of TiO_2_, then interact with the ambient oxygen (O_2_) and water (H_2_O) molecules. Holes oxidizes H_2_O molecules into highly reactive hydroxyl radicals (superoxide radical anion (O_2_^−⋅^), which is further reduced to OH^⋅^. Since these radicals are highly reactive, thus known as reactive oxygen species (ROSs). These formed ROSs on the surface of TiO_2_ react with the viruses and result into its degradation to CO_2_ and H_2_O^33^, as shown in the proposed schematic diagram of Fig. 6. Photocatalysis is a surface phenomenon, which oxidizes/reduces or degrades the organic pollutants. Therefore, the TiO_2_/Ti photocatalyst coating balls with a large specific surface area are easy to use, showing high environmental purification and virus inactivation functions.

## Conclusion

In present work, the TiO_2_/Ti photocatalyst coatings has been formed on Al_2_O_3_ balls using a simple and effective approach of mechanical coating method and followed oxidation in air. After oxidation in air, the larger amount of anatase TiO_2_ forms on the surface of Ti coatings, confirmed with XRD, SEM and XPS results. TiO_2_/Ti photocatalyst coatings on Al_2_O_3_ balls are effective for environmental purification, owing to their high decomposition function for C_2_H_4_O and CH_2_O. Notably, TiO_2_/Ti photocatalyst coatings also show significant viral inactivation capability, reaching 99.96% inactivation rate for influenza virus and 99.99% inactivation rate for new coronavirus.

## Supplementary Information

Supplementary Figures.

Supplementary Information.

## Data Availability

The data that support the findings of this study are available from the article and Supplementary Information files, or from the corresponding authors upon reasonable request.
