# Supplementary material for: Inactivation of SARS-CoV-2 and photocatalytic degradation by TiO2 photocatalyst coatings
Source: Sci Rep. 2022 Sep 26;12:16038. doi: 10.1038/s41598-022-20459-2 (PMC9512902; doi:10.1038/s41598-022-20459-2)
Supplement: Supplementary file 1 — Supplementary Figures. [file 41598_2022_20459_MOESM1_ESM.doc]

**Inactivation of SARS-CoV2 and** **photocatalytic degradation by TiO_2_ photocatalyst coating balls**

Yun Lu^a,1,*^, Sujun Guan^b,1^, Liang Hao^c^, Hiroyuki Yoshida^d^, Shohei Nakada^a^, Taisei Takisawa^a^, Takaomi Itoi^a^


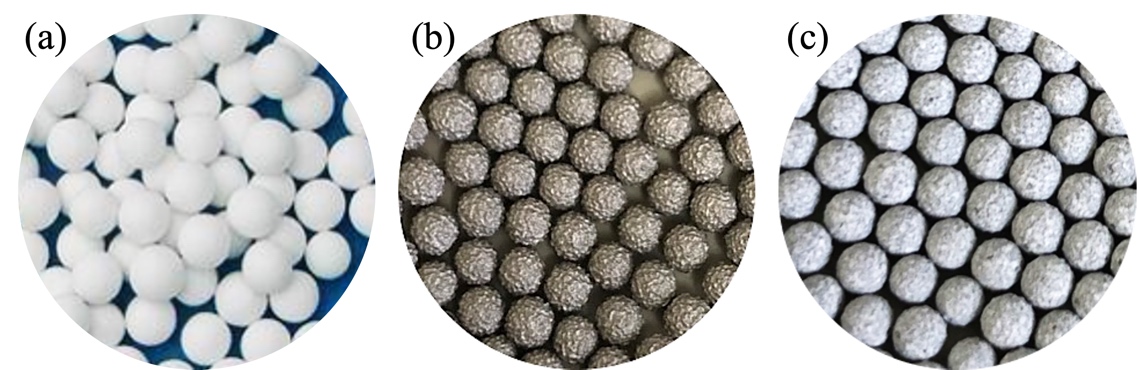


**Fig.S1** Appearance photographs of Al_2_O_3_ balls, Ti coating balls and TiO_2_ coating balls by MCT. (a) Al_2_O_3_ balls, (b) Ti coatings on Al_2_O_3_ balls, (c) TiO_2_/Ti coatings on Al_2_O_3_ balls.


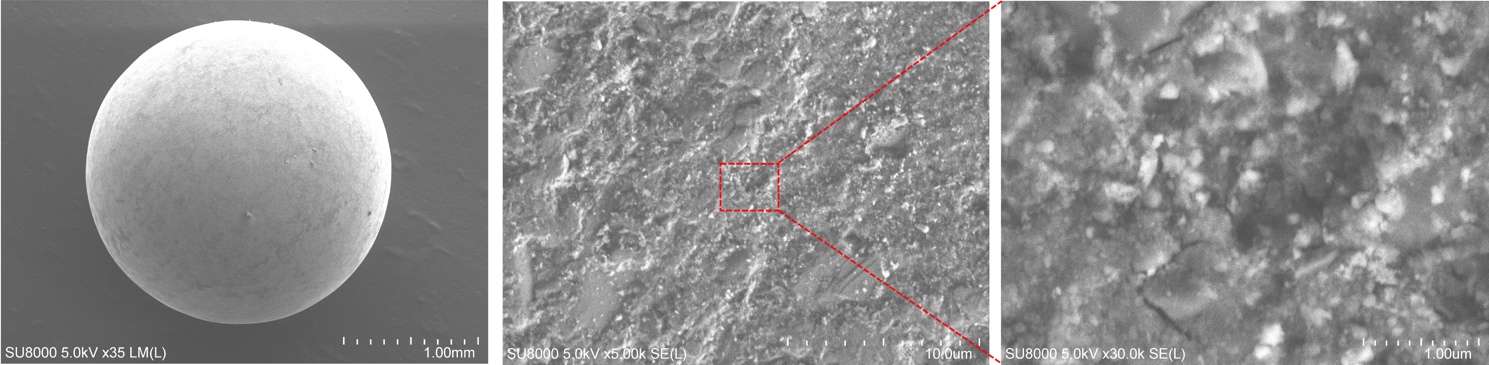


**Fig. S2** Surface morphology of of and Al_2_O_3_ ball. (Diameter: 2mm).
